# Transcriptional Regulation in Bacteria

**DOI:** 10.3390/microorganisms12122514

**Published:** 2024-12-06

**Authors:** Tomohiro Shimada

**Affiliations:** School of Agriculture, Meiji University, 1-1-1 Kawasaki-Shi, Kanagawa 214-8571, Japan; tomoshimada@meiji.ac.jp

## 1. Introduction

The goal of research in the post-genomic era, now that the full extent of the genes encoded on microbial genomes is known [https://www.ncbi.nlm.nih.gov/genome (accessed on 5 November 2024)], is to elucidate the whole transcriptional mechanism by which microorganism express genes through transcriptional regulation with transcriptional regulatory factors. The genome sequence of microorganisms reveals their gene set and the set of transcriptional regulators that regulate the expression of those genes, providing a complete overview of the transcriptional regulatory mechanisms in microorganisms [1,2]. Before genome sequencing was available, the analysis of the transcriptional regulation of transcriptional regulators occurred at the gene level, whereas it is now possible to analyze this at a genome-wide scale [3,4,5]. To understand the intrinsic transcriptional regulatory mechanisms, it is necessary to note that transcriptional regulation forms a hierarchical network structure and to understand the distinction between direct regulation and indirect effects [6,7,8,9,10,11].

The new Special Issue, entitled “Transcriptional Regulation in Bacteria”, in the *Microorganisms* journal, includes a total of ten original articles, providing new information about the transcriptional regulation of various functions in a wide variety of microorganisms. Recent studies were collected, ranging from detailed and precise studies of the transcriptional regulatory mechanisms of a single gene or via a single transcription factor [contributions 1–3] to studies of the genome regulatory networks of whole genomes and newly revealed transcriptional regulations [contributions 4–7]. It is also revealed that transcription factors comprehensively regulate multiple genes involved in multiple seemingly unrelated biological functions [contributions 8–10]. The analysis of transcriptional regulation revealed not only the molecular mechanisms of transcriptional regulation, but also the functional network of genes, leading to the elucidation of new biological functions of these microorganisms.

## 2. An Overview of Published Articles

Transcriptional regulation in microorganisms is carried out via the interaction of the transcription machinery, RNA polymerase, with the sigma subunit and transcription factors. Therefore, an elucidation of the function of functionally unknown transcription factors will provide new insights into novel transcriptional regulatory mechanisms and the environmental adaptation of microorganisms. Saito et al. identified a genomic binding site of a functionally unknown transcription factor, YegW, in *Escherichia coli.* Moreover, they showed that the effector is ADP–glucose, a precursor to glycogen synthesis, and that it functions as a repressor of the *yegTUV* operon, which is involved in glycogen accumulation. Its demonstrated effects on glycogen accumulation revealed its mechanism for accumulating a carbon source suitable for cell proliferation, and YegW was renamed GgaR (repressor of glycogen accumulation) [contribution 1]. Zhang et al. analyzed the *Bacillus thuringiensis* function unknown transcription factor HD73_5014 and found that it increased the transcript level of *pepV*, which encodes a dipeptidase. They proposed that HD73_5014 is renamed PepR (dipeptidase regulator) [contribution 2]. Belin et al. analyzed the interaction of the arabinose-responsive transcription factor AraC of *Escherichia coli* with the α subunit of RNA polymerase in detail. It became clear that the N-terminal domain of AraC exhibits at least three distinct activities: dimerization, arabinose binding, and transcriptional activation [contribution 3]. These research results focus on the functions of the transcription factors, revealing new physiological roles of transcription factors and new molecular mechanisms of transcription factors.

The function of a transcription factor has often been understood from the perspective of the function of a specific target gene(s). Thus, even for transcription factors thought to have a known function, their precise role in the genome as a whole remains unknown. Therefore, once again, a genome-wide comprehensive analysis could contribute to the elucidation of new target gene(s) and their precise role. Xiao et al. analyzed the culture conditions under which PurR, a known transcription factor for purine biosynthesis in *Yersinia pestis*, is affected, as well as providing a quantitative RNA analysis. They found novel targets, including the type VI secretion system, and discovered a new physiological role for PurR [contribution 4]. In *Acinetobacter baumannii*, Huang et al. analyzed EmaSR, a two-component regulatory system of ethanol metabolism, and clarified its role in ethanol and the acetate metabolism, as well as its regulation of the acetate:succinyl-CoA transferase gene [contribution 5]. Huang et al. analyzed the mechanism by which the two-component regulatory system LiaSR induces Chlorhexidine resistance in *Streptococcus mutans*, suggesting that this resistance ability is achieved via the regulation of the *lmrB* efflux pump [contribution 6]. Xu et al. analyzed HigBA2, one of the TA systems in the main persister formation factors in *Mycobacterium tuberculosis*, and identified a novel set of target genes based on the HigA2-binding motif. This provided insights into the mechanism of parsister formation [contribution 7]. This research reveals a new transcriptional regulatory network based on a genome-wide analysis of transcription factors whose functions were previously known. Approaches such as these are useful not only for understanding the intrinsic role of transcription factors in the genome, but also for understanding how microorganisms adapt to changes in the environment.

To understand the molecular mechanisms of phenomena in which the involvement of specific transcription factors is unknown, approaches that analyze gene expression across the genome, such as transcriptome analysis, are useful. Song et al. studied the mechanism of feather degradation by *Pseudomonas aeruginosa* Gxun-7 for bioremediation. The RNA-seq analysis inferred a set of genes involved in feather degradation and proposed a model for the molecular mechanism [contribution 8]. Sanders et al. analyzed the mechanism of silver ion resistance in *Escherichia coli*. They identified a group of genes that responded to ionic silver and revealed that two-component regulatory systems such as CusSR and HprSR were involved in the major response [contribution 9]. Schlüter et al. improved acarbose yield by regulating the expression level of osmotic stress sigma factor σH^As^ in *Actinoplanes* sp. SE50/110. Transcriptome analysis identified a set of genes affected by σH^As^ and contributed to our understanding of its genome-wide role [contribution 10]. These approaches identified the transcriptional regulators involved in these important phenomena.

## 3. Conclusions

This Special Issue addresses the understanding of diverse transcriptional regulatory mechanisms in various microbial species. Although the genome sequences of microorganisms are rapidly being revealed, experimental analyses of individual transcriptional regulatory mechanisms are necessary because these sequences do not tell us how, when, and to what extent genes are expressed [12,13,14]. In addition, although the recent research tends to emphasize the analysis of some genes that directly benefit human society, it is necessary to understand the regulatory mechanisms of the entire genome because the genes of an organism form a network structure and multiple groups of genes function in a coordinated manner [15,16]. One of our major goals is to understand the entire transcriptional regulatory mechanism based on the functional elucidation of all transcriptional regulators in a single organism.
